# Contaminated Small Drinking Water Supplies and Risk of Infectious Intestinal Disease: A Prospective Cohort Study

**DOI:** 10.1371/journal.pone.0042762

**Published:** 2012-08-24

**Authors:** Helen L. Risebro, Lynette Breton, Heather Aird, Alan Hooper, Paul R. Hunter

**Affiliations:** 1 Norwich Medical School, University of East Anglia, Norwich, United Kingdom; 2 Food Water and Environment Laboratory, The Norfolk and Norwich University Hospital NHS Trust, Norwich, United Kingdom; 3 Department of Microbiology and Immunology, Hereford County Hospital, Hereford, United Kingdom; Public Health Agency of Barcelona, Spain

## Abstract

**Background:**

This study sought to identify whether elevated risk of infectious intestinal disease (IID) exists in contaminated small water supply consumers compared with consumers drinking from small supplies complying with current standards and whether this effect is modified by age.

**Methodology and Principal Findings:**

A prospective cohort study of 611 individuals receiving small supplies in England was conducted. Water supplies received sanitary inspection and examination for indicator bacteria and participants maintained a daily record of IID. Regression modeling with generalized estimating equations that included interaction terms between age and indicators of fecal pollution was performed. Crude IID prevalence was 9·3 days with symptoms/1000 person days (95%CI: 8·4, 10·1) and incidence was 3·2 episodes/1000 person days (95%CI, 2·7, 3·7) or 1·2 episodes per person year. Although there was no overall association between IID risk and indicator presence, there was strong interaction between age and indicator presence. In children under ten, relative risk (RR) of IID in those drinking from enterococci contaminated supplies was 4.8 (95%CI: 1.5, 15.3) for incidence and 8.9 (95%CI: 2.8, 27.5) for prevalence. In those aged 10 to 59, IID risk was lower but not statistically significant.

**Conclusions:**

Contaminated small water supplies pose a substantial risk of IID to young children who live in homes reliant on these supplies. By contrast older children and adults do not appear to be at increased risk. Health care professionals with responsibility for children living in homes provided by very small water supplies should make parents aware of the risk.

## Introduction

In 2009 the committees on environmental health and on infectious diseases of the American Academy of Pediatrics published a technical report entitled “Drinking water from private wells and risks to children” [1]. In that report the authors quite rightly raised concerns about the risks to children's health from both microbiological and chemical agents when they take their drinking water from very small water supplies, especially from wells. However, as pointed out by the authors, they were not able to draw on any substantial body of epidemiological evidence to support the concerns raised for microbiological threats. The study reported in this paper was designed to provide further quantitative evidence of the risks from such supplies to all consumers but children especially.

Small water supplies may be derived from a well, borehole, spring or surface water, though in England surface water supplies tend to be less common. Throughout Europe most very small supplies are owned and managed by the consumer. In the UK these small supplies are often referred to as private water supplies (PWS) and some 50,000 PWS serve an estimated 1% of the UK population [2]. However many more people will consume water as visitors.. Although the large majority serve single dwellings, some PWS can be large and supply many properties [3]. In Europe, one in ten people are supplied by small water systems [4]. In the United States waters are not classified in the same way, but an estimated 125,126 very small water systems (supplying 25 to 500 people) provide water to over 14 million people with many more supplies serving less than 25 people [5]. In North America reliance on very small water supplies is a particular issue for Native Americans [6].

In Europe small water supplies are governed by the Council Directive 98/83/EC of 3 November 1998 on the quality of water intended for human consumption [7]. In England this legislation was incorporated into The Private Water Supplies Regulations 2009 [8]. This legislation requires both Enterococci and *Escherichia coli* (indicators of fecal pollution) to be absent in 100 ml. However, the presence of indicators in PWS throughout the UK is well documented [9]–[13], and it is estimated that 54% of PWS are non-compliant with regulations [12].

Outbreaks of disease linked to PWS are common. Between the years 1970–2000, 25 outbreaks of gastrointestinal illness (36% of all drinking water outbreaks) were associated with PWS in England and Wales causing 1584 cases of illness and putting 5190 people at risk [13]. However, it is probable that substantial under ascertainment exists and that most disease burden is associated with sporadic disease which is even more difficult to quantify.

To-date there have been three prospective cohort studies that have attempted to associate sporadic and endemic gastrointestinal illness with the presence of faecal indicator bacteria in water at the tap in developed countries, one in France[14] and two in Canada [15],[16] as well as a retrospective case-control study in the US [17]. The two Canadian studies found an elevated risk of gastrointestinal illness in people drinking from contaminated supplies but were insufficiently powered to detect statistically significance associations (OR 1·52; 95% CI: 0·33, 6·92) [15], and (OR, 2·11; 95% CI: 0·90, 4·94) [16]. The French study did find a statistically significant increased risk but focused on water supplies to larger villages rather than very small supplies (RR, 1·36; 95% CI: 1·24, 1·49) [14]. More recently, a case-control study of septic tank density and diarrhea found a risk of illness six times greater in children (aged 1 to <19 years) drinking from Enterococci contaminated wells in the US (Adjusted OR, 6·18; 95% CI: 1·22, 31·46) [17]. However, retrospective case control studies are comparatively more likely to produce bias than prospective studies and, coupled with the aforementioned limitations of the prospective studies, it is still not possible to be definitive about the disease burden attributed to contaminated small supplies and how this effect may vary with age. The study reported here was designed to investigate whether an elevated risk of infectious intestinal disease (IID) exists in individuals who consume water from contaminated small supplies compared with those who drink from small supplies that comply with current standards and also whether this effect is modified by age.

## Materials and Methods

We conducted a prospective cohort study of PWS consumers in two rural areas of England: East Anglia (Norfolk and Suffolk) and Herefordshire. Participants completed a 12 week symptom diary and two water samples measured exposure. This reseach project was reviewed and approved by the Norfolk Research Ethics Committee REC reference number: 07/H0310/138. Written informed consent was obtain from all participants or, in the case of children, from a parent or guardian.

### Recruitment & data collection

A random sample of 2539 households of approximately 6000 believed to be served by a private supply was sent a postal questionnaire with the option of participating in the cohort study. Interested households were recruited on a rolling monthly basis to account for seasonal variations in water quality or disease risk [12]. In Norfolk and Suffolk, recruitment occurred between January and December 2008 and follow-up completed in March 2009. In Herefordshire, recruitment occurred between October 2008 and September 2009 and follow-up completed in January 2010.

Household visits were carried out by two trained field researchers. Individual and household level data was collected via structured interviewer- and self-administered questionnaire. Where possible, supply and treatment systems were inspected to gather data on supply characteristics and a trundle was used to measure distance between source and septic tank. Participants reported their perception of water quality at baseline to determine risk perception bias in self-reported illness [18].

### Infectious intestinal disease

Participants completed a daily diary for 12 weeks capturing occurrence of: diarrhoea, diarrhoea with blood, vomiting, stomach pain/cramps, nausea, headache, high temperature/fever, cough, runny/blocked nose or sore throat, no symptoms and overnight travel (abroad and UK). Participants recorded the number of times vomiting and/or diarrhoea occurred in a 24 hour period. Households received a weekly telephone call to collect and record diary data. In general the senior female in the house was the person who reported on the contents of the diary, though this varied from house to house and from week to week.

We defined a case of IID as diarrhoea once or more in a 24 hour period and/or vomiting more than once in a 24 hour period. This is similar to the case definition used by the two UK IID studies [19], [20] However, unlike in the UK IID, we did not exclude cases lasting more than two weeks as duration of illness for cryptosporidiosis, a potentially important illness, is frequently in excess of this [21]. We also left it to the participants to decide for themselves what was diarrhoea. The definition of a new incident of IID (or ‘episode’) was any day satisfying the above definition but preceded by six symptom free days as used previously [22]. The first six days of data collected from each individual were excluded to meet the new incident definition. We used both incidence and prevalence as outcome measures as with other authors' recent work [22]. Incidence was defined as the number of new incidents and prevalence as the number of days with diarrhoea in a given time period. We consider the use of prevalence has the benefit of being a better indicator of disease burden.

### Exposure measurement

Exposure was measured through collection of two 500 ml samples from the principal drinking water tap (at recruitment and 12 weeks post recruitment). The tap was sterilized and run for 2 minutes minimum prior to sample collection. Samples were transported under refrigeration to reach the laboratory within six hours.

Water samples were analyzed by the Norfolk and Norwich University Hospitals NHS Foundation Trust (NNUH NHSFT) Food, Water and Environmental (FW&E) Microbiology Laboratory and the FW&E laboratory at Hereford County Hospital. Water samples were analyzed by the IDEXX Quanti-tray method (HPA Standard Method W18 Enumeration of Coliforms and Escherichia coli by IDEXX (Colilert 18) Quanti-tray™) for Coliforms and *E. coli* and Enterococci by membrane filtration (HPA standard method W3 Enumeration of Enterococci by Membrane Filtration). The NNUH NHSFT FW&E laboratory was accredited by the United Kingdom Accreditation Service (UKAS) during the study period. The Hereford laboratory was UKAS accredited until March 2009 and subsequently accredited by the Campden Laboratory Accreditation Scheme (CLAS).

The telephonist and participants were blind to results of the first water sample throughout the 12 week observation period unless *E. coli* and/or Enterococci were detected at or greater than 100/100 ml. If coliforms, *E. coli* or Enterococci were present in either of the two samples, the supply was defined as contaminated by the indicator. If participants were told of their results prior to completion of the data collection, they were excluded from further analysis.

### Sample size

Based on a conservative estimate that 20% of supplies would contain indicator organisms [12], and the estimated incidence of IID to be 0·25 per 12 weeks [15],[22], we estimated (using Signorini method for Poisson regression with a variance inflation factor to allow for within-household correlation [23], a sample size of 144 would be sufficient to detect a relative risk of 2.8 (power = 0.80; alpha level = 0.05). Because early water testing results from the first study region showed lower exposure than predicted we extended the study to a second region applying the same sample size.

### Statistical analysis

Data analysis was performed in PASW™ version 18 (previously SPSS). For associations between diarrhoea incidence/prevalence, Generalized Estimating Equations (GEE) were used with study centre (East Anglia or Herefordshire), household and individual as subject variables. GEE are an extension of the generalized linear model for use where unknown correlation is present [24]. The approach is particularly suited for situations with multiple responses from the same participant or where participants' responses may be correlated such as when they are members of the same family, as in this study. Missing values were not replaced. A Poisson loglinear model was fitted using the GEE method to derive relative risks. Initially all key predictor variables and possible confounders were tested as single variable predictors. For the final model all variables statistically significant at the p = 0·2 level were included and the least statistically significant variable removed until all remaining variables were statistically significant at the p = 0·2 level. We did not replace missing values. Participants without a second water sample were excluded. Participants completing less than six diary days were included in prevalence but excluded from incidence calculations.

## Results

**Figure 1:**
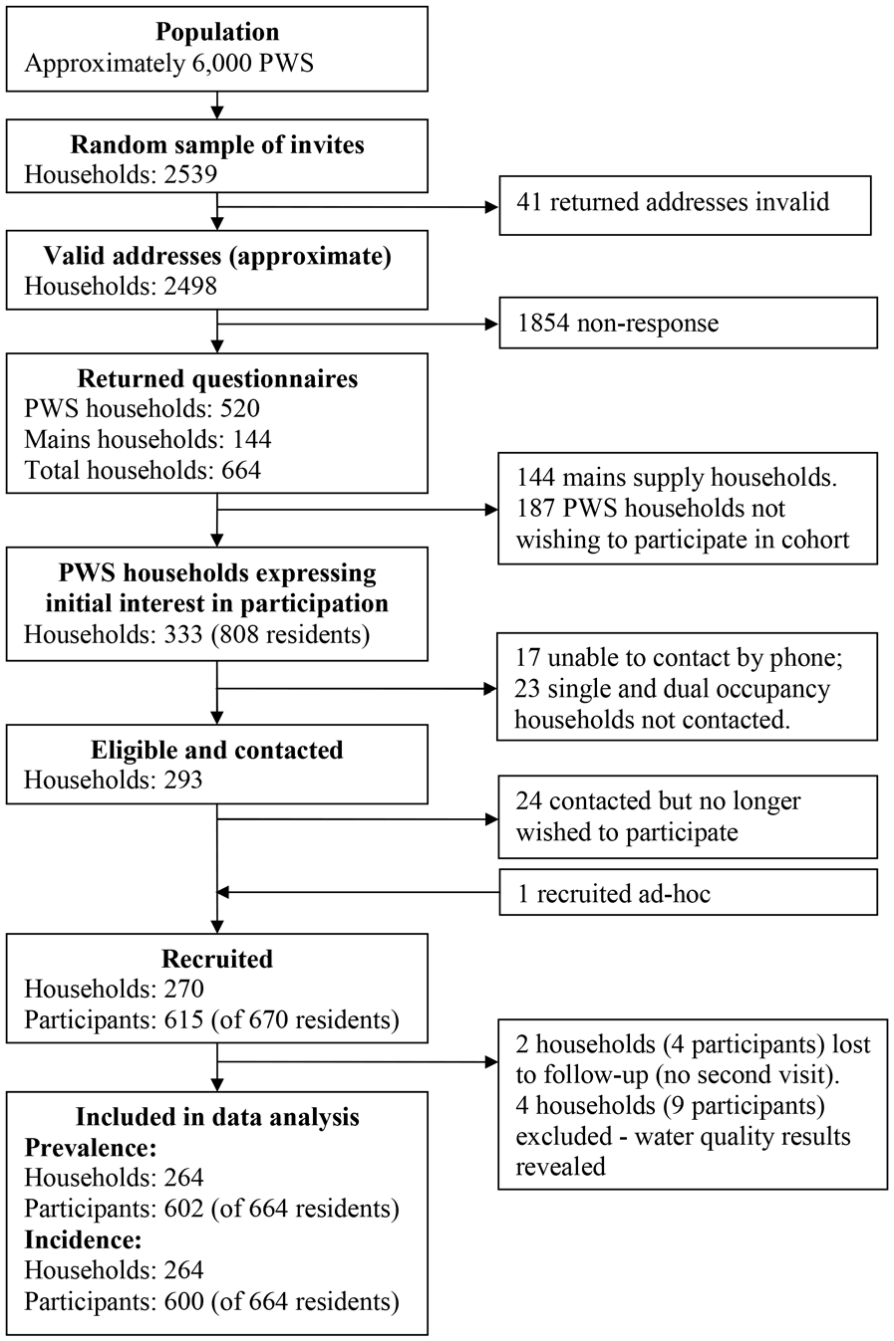
Flow Chart of Participant and Household Selection and Recruitment, England, 2008–2010.

The recruitment flow chart is shown in Figure 1. The overall questionnaire response rate was 27% (664/2498). Of the 664 responses, 144 were received from households supplied by mains water and 520 from households with private supplies, 333 of the 520 wished to participate in the cohort study. Attempts were made to contact all households with three or more residents and a random selection of the remainder due to the over-representation of homes with only one or two residents. Of the 293 eligible households contacted, 269 (92%) agreed to participate plus one additional household neighbouring a participant. Of the 670 residents at these properties, 615 agreed to participate (92%). Two households did not complete the final visit and four households whose water quality results were revealed (on account of exceeding the 100 *E. coli* and/or Enterococci per 100 ml limit) before the end of the data collection were excluded, leaving 264 households, with 602 participants for prevalence analysis. Furthermore, two participants completed less than 6 diary days, leaving 600 participants for incidence analysis. Ninety-one percent (550/602) of participants completed the full 84 diary days (12 weeks). Altogether there were 49,811 and 46,207 person days of observation for prevalence and incidence calculations respectively. Tables 1 and 2 report the household and individual baseline characteristics respectively.

**Table 1:** Baseline Characteristics of Households with PWS, England, 2008–2009.

Characteristic	No. of households (N = 268 except where indicated)	%
**Region**		
Norfolk/Suffolk	135	51
Herefordshire	129	49
**All residents in household**		
1	31	12
2	148	56
3	34	13
4	33	13
5	14	5
6	4	2
**Employment skill level of main earner** a		
1	4	2
2	12	5
3	69	26
4	71	27
Retired	102	39
Unemployed	0	0
Other	2	0.8
Missing	4	2
**Maximum household education**		
Degree	171	65
Further Education	37	14
Minimum school leaving age	22	8
Missing	34	13
**Ownership of animals**		
Pets	201	76
Livestock	101	38
None	53	20
Missing	0	0
**Length of time living at residence**		
0 to 9 years	88	33
10 to 19 years	84	32
20 to 29 years	49	19
30+ years	42	16
Missing	1	0.4

Characteristic	No. of households (N = 264 except where indicated)	%
**Water Source**		
Borehole	153	58
Spring	33	13
Well	75	28
River/Stream	1	0.4
Other/Unknown	2	0.8
**No. of households served by supply**		
1	197	75
2	39	15
3	12	5
4+	16	6
**Treatment (point of use or entry)**		
Any treatment (filtration, chlorination, UV)	100	38
- Chlorination n = 100	3	1
- Filtration (e.g. reverse osmosis) n = 100	85	32
- UV n = 100	52	20
No treatment	161	61
Unknown	3	1
**Details of treatment maintenance** n = 100		
Recorded	52	52
Not recorded	35	35
Unknown	13	13
**Sewerage system**		
Onsite sewerage	245	93
Mains sewerage	18	7
Missing	1	0.4
**Distance septic tank from source** n = 245^b^		
0–19 m	46	19
20–39 m	76	31
40–59 m	35	14
60 m+	62	25
Missing	26	11
**Depth of well** n = 75^c^		
0–4 m	19	25
5–9 m	18	24
10–14 m	5	7
15 m+	9	12
Missing/Unknown	24	32
**Depth of borehole** n = 153^c^		
0–29 m	30	20
30–59 m	55	36
60–89 m	14	9
90 m+	8	5
Missing/Unknown	46	30

a Occupations coded according to SOC2000 (Office for National Statistics 2000). Occupations in skill level 1 require general education, 2 require good general education/work experience, 3 require post compulsory education and 4 require a degree or equivalent work experience.

b Forty-six percent (72/158)measured by a trundle, 54% (85/158)estimated by the researcher.

c Based upon an estimate provided by the householder.

**Table 2:** Baseline Characteristics of Participants with PWS, England, 2008–2009.

Characteristic	Number of participants (n = 602, except where indicated)	%
**Male**	289	48
**Age Group**		
<10	39	6
10–29	76	13
30–59	246	41
60+	241	40
**Chronic intestinal illness**		
Any reported chronic intestinal illness	51	8
- Diagnosed by clinician n = 51	27	53
- Self-reported n = 51	19	37
- Missing n = 51	5	10
No chronic intestinal illness reported	528	88
Missing	23	4
**Currently taking Medication**		
Taking antibiotics, steroids or antacids	69	11
Not taking antibiotics, steroids or antacids	500	83
Missing/unknown	38	6
**Water Consumption from PWS**		
Report drinking from PWS	582	97
Report drinking no water from PWS	6	1
Missing	14	2
**Cold water consumption from PWS/glasses**		
0	65	11
1–5	437	73
6–10	72	12
11–15	4	0.7
Missing	24	4
**Boiled water consumption from PWS/glasses**		
0	70	12
1–5	313	52
6–10	183	30
11–15	7	1
16–20	3	0.5
Missing	26	4
***“Generally my tap water is good”*** d		
Completely disagree, disagree a lot, disagree	17	3
Neither agree nor disagree	23	4
Completely agree, agree a lot, agree	502	83
Missing	60	10

d Seven point likert scale response to question, results aggregated.

### Water sampling

Table 3 shows water quality results of 568 samples taken from 268 PWS. As mentioned, blinding to water quality was unsuccessful for 4 households whose first set of results met or exceeded 100 *E. coli* and/or Enterococci per 100 ml, and these 4 households were excluded from further analysis.

**Table 3:** PWS Water Quality Results by Supply and Sample, England, 2008–2010.

Indicator Bacteria	Supplies with indicator in either sample (N = 268)	%	Samples with indicator present (N = 536)	%
Coliforms	126	47	198	37
*E. coli*	63	24	85	16
Enterococci	75	28	102	19
Any of the 3 indicators	128	48	202	38

### Risk of infectious intestinal disease

**Figure 2:**
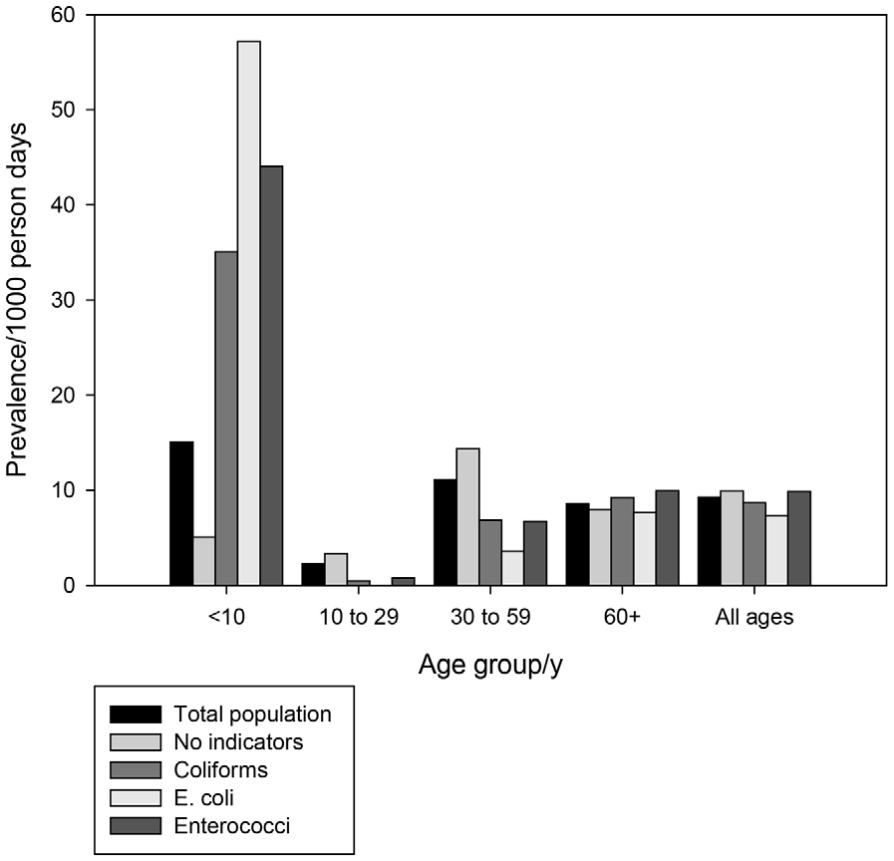
Daily Prevalence of IID by Age Group and by Presence of Indicator Organisms in Water, England, 2008–2010.

**Figure 3:**
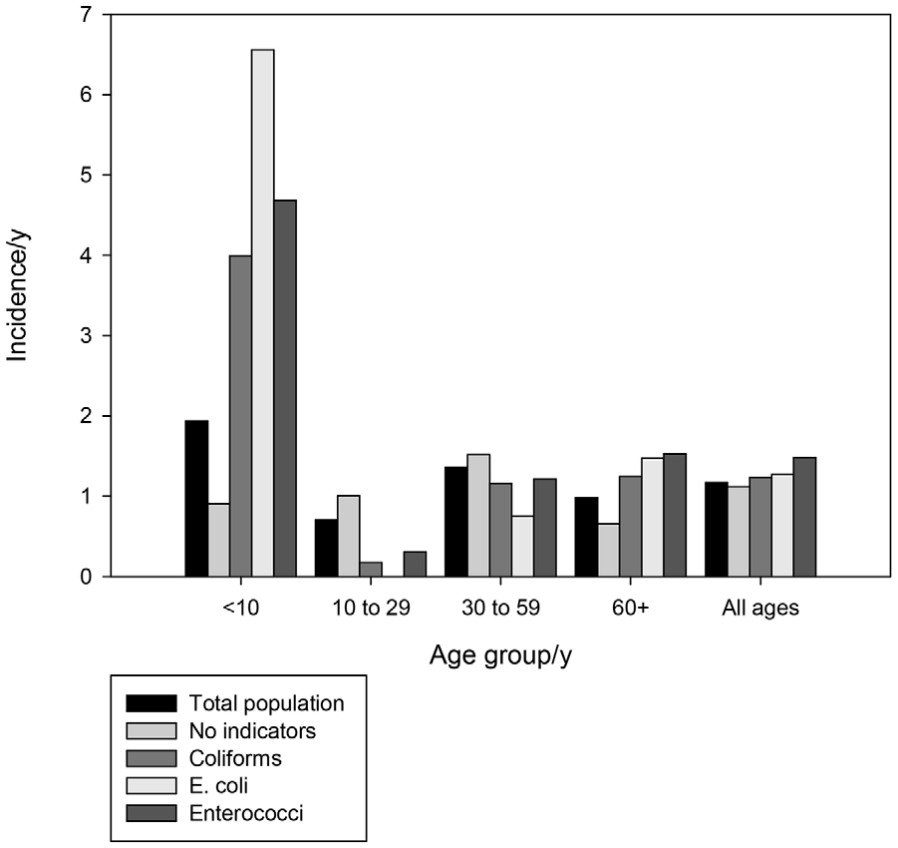
Annual Incidence of IID by Age Group and by Presence of Indicator Organisms in Water, England, 2008–2010.

The calculated crude IID prevalence was 9·3 days with symptoms/1000 person days (95%CI: 8·4, 10·1). The crude incidence was 3·2 episodes/1000 person days (95%CI: 2·7, 3·7) or 1.2 episodes per person year. Figures 2 and 3 illustrate daily prevalence and annual incidence by age group in regard to whether or not faecal indicator bacteria were detected in the supply. Of particular note is the high incidence of diarrhoea in children under 10 in homes provided by water with Enterococci present, 4·8 episodes per person per year (95%CI: 2·2, 8·6).

The association between covariates and risk of illness are shown in Table 4. For disease incidence as the outcome variable, the principle earner being retired was negatively associated with disease risk (Relative Risk (RR) = 0.58, 95% Confidence Intervals (CI): 0.35, 0.95). For prevalence as the outcome variable, increased risk was associated with having an onsite sewage disposal system (RR = 3.08, 95%CI: 1.45, 6.52) and negatively associated with the male gender (RR = 0.53, 95% CI: 0.28, 1.00) and increasing glasses of unboiled private water (RR = 0.87, 95%CI: 0.77, 0.98).

**Table 4:** Association between risk of IID and possible confounding variables in participants with private water supplies.

Variable		Prevalence		Incidence	
		Relative Risk	95% Confidence Interval	Relative Risk	95% Confidence Interval
Pets	No	1		1	
	Yes	1.27	0.61–2.63	1.13	0.63–2.02
Livestock	No	1		1	
	Yes	0.70	0.36–1.26	0.86	0.56–1.32
Sewage system	Mains	1		1	
	On site	3.08	1.45–6.52	1.64	0.80–3.37
Ownership of private supply	Other	1		1	
	Householder	0.81	0.31–2.08	0.90	0.48–1.71
Overseas travel in previous two weeks	No	1		1	
	Yes	1.18	0.44–3.17	0.94	0.47–1.86
Maximum educational level in household	Secondary	1		1	
	Post secondary	2.41	0.89–6.54	2.68	0.83–8.72
Employment status of main earner	Employed	1		1	
	Retired	0.98	0.47–2.04	0.58	0.35–0.95
Employment skill level	/level increment	1.14	0.63–2.09	1.18	0.79–1.76
Water source	Borehole	1		1	
	Well	1.24	0.65–2.37	1.22	0.78–1.93
	Surface/spring	1.17	0.35–3.89	0.88	0.38–2.06
Water treatment	No	1		1	
	Yes	0.96	0.50–1.84	1.16	0.74–1.79
Residents in household	/resident	0.78	0.56–1.09	1.12	0.94–1.33
Length of time at residence	/years	0.99	0.97–1.02	0.99	0.96–1.02
Distance of septic tank from source	/100 m	1.00	0.89–1.11	1.06	0.98–1.16
Properties using supply	/property	1.00	0.98–1.02	1.01	0.99–1.02
Borehole or well depth	/m	1.01	1.00–1.03	1.00	0.99–1.01
Gender	Female	1		1	
	Male	0.53	0.28–1.00	0.64	0.41–1.00
Quantity unboiled water from private supply	/glass	0.87	0.77–0.98	0.93	0.83–1.04
“Generally my tap water is good”^a^	/category	1.01	0.78–1.31	0.90	0.77–1.04

a Seven point likert scale response to question, where 1 represents ‘completely disagree’ through to 7 representing ‘completely agree’.

Table 5 shows the unadjusted relative risks for IID both prevalence and incidence unadjusted and adjusted for log age and the interaction term between log age and indicator presence. Considering each indicator independently, it can be seen that none of the indicators were associated with IID prevalence or incidence. There was an elevated incidence of IID for individuals drinking from a supply contaminated with Enterococci but this was not statistically significant (RR = 1.41, 95% CI: 0.88, 2.26, p = 0.152). However, in the models with log age as an interactive term, there was a statistically significant effect of both *E. coli* and Enterococci on IID prevalence but not incidence. Subgroup analyses based on age group identified the presence of Enterococci and/or *E. coli* as having a statistically significant impact on risk of IID prevalence and incidence in children under ten years old. There was also an impact of Enterococci on disease incidence in people over 60 years (RR = 1.97, 95%CI: 0.92, 4.25) but this association did not reach statistical significance (p = 0.08). The goodness of fit for the models with *E. coli* and Enterococci were similar and better than the model with coliform.

**Table 5:** Prevalence and Incident Rate Ratios of IID, England, 2008–2010.

	Daily prevalence		Incidence	
Single indicator	Relative Risk	95% Confidence Interval	Relative Risk	95% Confidence Interval
Any coliform	0.88	0.47–1.64	1.11	0.71–1.71
Any *E. coli*	0.72	0.37–1.40	1.10	0.62–1.95
Any Enterococci	1.09	0.59–1.99	1.41	0.88–2.26
*E. coli* and or Enterococci	0.96	0.52–1.76	1.25	0.78–2.00
**Interaction with log age**				
Log age	2.67	0.86–8.22	0.96	0.51–1.83
Any coliform	13.9	1.19–162.9	2.69	0.30–20.8
Any coliform*Log age	0.18	0.04–0.91	0.58	0.17–2.06
Log age	2.01	0.77–5.26	0.88	0.52–1.49
Any *E. coli*	18.4	1.39–242.4	3.01	0.15–59.8
Any *E. coli* *Log age	0.14	0.03–0.66	0.55	0.09–3.26
Log age	2.55	0.92–7.07	0.92	0.51–1.66
Any Enterococci	25.3	2.490–256.8	3.41	0.39–29.68
Any Enterococci*Log age	0.14	0.03–0.63	0.58	0.15–2.21
Log age	2.60	0.93–7.23	0.94	0.52–1.69
Any fecal indicator	24.2	2.33–251.7	3.23	0.36–28.95
Any fecal indicator*Log age	0.14	0.03–0.61	0.56	0.14–2.16
**Any Enterococci by age group**				
Age<10	8.85	2.85–27.5	4.78	1.50–15.29
Age 10 to 29	0.31	0.04–2.34	0.40	0.06–2.93
Age 30 to 59	0.58	0.24–1.41	0.91	0.44–1.85
Age 60+	1.13	0.43–2.97	1.97	0.92–4.25
**Any ** ***E. coli*** ** by age group**				
Age<10	6.47	1.89–22.2	5.24	1.36–20.18
Age 10 to 29	Not estimable		Not estimable	
Age 30 to 59	0.30	0.10–0.88	0.51	0.18–1.40
Age 60+	0.73	0.28–1.89	1.75	0.79–3.86
**Any fecal indicator by age group**				
Age<10	8.85	2.85–27.5	4.78	1.50–15.29
Age 10 to 29	0.24	0.03–1.87	0.32	0.04–2.34
Age 30 to 59	0.52	0.21–1.27	0.81	0.40–1.67
Age 60+	0.99	0.38–2.60	1.73	0.80–3.73

Prevalence and incidence given by indicator (with/without), including interaction terms with log age and by age group. Referent group for indicator bacteria is supplies with indicator absemt.

For disease prevalence all possible confounding variables (those with p<0.2) were then included with presence of Enterococci and log age with interaction terms in a Poisson GEE regression model. These included onsite sewage disposal, number of participants in home, quantity of unboiled private supply water and gender. After the GEE model was run the least statistically significant variable was removed from the model and the model re-run until all variables in the model were statistically significant at the *P* = 0·2 level or greater. The final model is shown in Table 6. The presence of Enterococci with log age as an interactive term remains statistically significant. Also positively associated was the presence of onsite sewage disposal (RR = 3.32; 95%CI: 1·42, 7.74) and someone in the house having attended post secondary school education (RR = 3.22; 95%CI: 1.11, 9.31). Negatively associated was the quantity of unboiled water drank from the private supply/glass (RR = 0.87; 95%CI: 0·78, 0.97).

**Table 6:** Best Fit Final Model for Prevalence of Intestinal Infectious Disease in People Living in Homes with Private Water Supplies, England, 2008–2010.

Variable	Parameter	Relative Risk	95% Confidence Interval
Any Enterococci	No	1	
	Yes	23.74	2.00–282.4
Log age		3.29	0.97–11.22
Any Enterococci * Log age		0.13	0.03–0.64
Sewage	Mains	1	
	Onsite	3.32	1.42–7.74
Gender	Female	1	
	Male	0.50	0.26–0.98
Quantity unboiled private supply water	/glass	0.87	0.78–0.97
Maximum education level in home	Secondary	1	
	Post secondary	3.22	1.11–9.31

## Discussion

This study provides important new evidence that contaminated small rural drinking water supplies are an important risk factor for IID. This study has found that the incidence of IID in people served by PWS is much higher than estimates of IID in the UK population as a whole. During a similar time period as this study, the UK IID2 study was also undertaken. UK IID2 reported an age and sex-standardised rate of 274 cases per 1,000 person-years (95% CI: 254 to 296), though when both definite and possible cases were included there were 523 cases per 1,000 person-years (95% CI: 497–551) [25]. People in our cohort reported an incidence some six fold greater than in the IID2 study. However, one should be cautious when interpreting this comparison. Our cohort was followed for only three months (not six), stool samples were not requested from cases and the case and episode definition differed slightly. Nevertheless, the incidence rates are still very different and unlikely to be explainable purely by these differences in study design.

Perhaps the clearest finding is that within consumers of PWS, IID varies between those for whom there is microbiological evidence of fecal pollution but only in certain age groups. Most importantly the main risk falls on children under ten for both prevalence and incidence. In the ten to 29 and 30 to 59 age groups disease risk is not greater in the exposed group and may even be lower. By contrast almost all of the excesss disease burden falls on children under 10 years old. Indeed, the incidence of IID in children under ten years with polluted water is not that dissimilar from those seen in many developing countries [26].

This impact of age on disease risk is in line with our prior work where we predicted that, for commonly encountered pathogens, overall population disease incidence may not vary much between populations with high and low exposure to infectious agents as a result of the development of immunity to commonly encountered pathogens [27]. The effect of repeat exposure in depressing population incidence may be even more pronounced for many enteric pathogens that have relatively short durations of immunity (6 months or so) [28]. However, whatever the impact on all age incidence young children always suffer more illness in higher exposure settings and as previously pointed out it is very young children that suffer the most in an episode of diarrhoeal disease with greater rates of hospitalization and higher mortality rates [29], [30].

Some covariates identified through the literature as having a potential for confounding were either not included in the model (at the cut-off limit of p<0.2) or not shown to have a statistically significant association (p<0.05) with risk of prevalence and incidence of IID. These covariates include ownership of pets and livestock, overseas travel, presence/absence of water treatment and, distance of septic tank from the source of the supply. Possible explanations include measurement error owing to the self-reported nature of observations (such as, ownership of animals and overseas travel), variation between householders in the accuracy of estimates provided (such as, distance from source to supply where distance was not measured with a trundle) or, in the case of treatment, systems which were potentially not functional and/or well-maintained.

One potential issue is the response and recruitment rates in our study (14.1% (333/2354) and 11.4% (269/2354) respectively) which used postal invite. These rates represent underestimates as it is not known how many of the 1854 non-responders received a mains supply (as opposed to PWS), yet they are still high compared to other recent cohorts conducted in the UK including IID2 (10.5% and 9% respectively) [25]. Also in the pilot phase of the UK Biobank study (a large and well funded cohort study) a recruitment rate of only 8% was achieved, 5% via phone and just 3% by post [31]. Although low recruitment rates may decrease statistical power to estimate the population disease burden, this should not bias our results or affect the primary outcome measure of this study, namely the relative risk between those people consuming water that is and is not fecally polluted.

Another issue is the use of self-report of IID. In this study we did not seek to determine the microbial cause of the diarrhoeal illness, partly because of concerns that requesting stool samples may suppress reporting of clinical illness [32]. The use of self-reported diarrhoea, a subjective assessment, could be prone to reporting bias if the respondent has a prior belief about the quality of the water supply. However, we have demonstrated that there was no relationship between respondents' beliefs about water quality and the presence of faecal indicators indicating that such bias is unlikely to play any effect. However, the different relative risk estimates for children under ten between incidence and prevalence rates would indicate longer duration of illness per episode in children, though this did not achieve significance. Unfortunately, the study was not sufficiently powered to undertake further analyses on the impact of duration of illness on the study findings. Longer durations of diarrhoea are particularly seen in cases of cryptosporidiosis and giardiasis, two pathogens commonly associated with PWS [3].

Because we were only able to take two samples, it is likely that there will have been some degree of exposure misclassification because of intermittent contamination. Some supplies were recorded as indicator negative when it is possible that had we taken more samples these would have been positive. Such misclassification would only have served to reduce the observed strengths of association.

In its report the American Academy of Pediatrics, recommended annual testing of well waters in homes with children [1]. We do not consider this sufficient. We have previously shown that there can be significant variation in results from one microbiological sample to another such that annual sampling would not be a sufficient predictor of risk, the probability of having at least one failed sample from a site has been shown to increase with the number of samples taken [12]. Instead we would argue that all parents reliant on very small drinking water supplies should either ensure adequate treatment or provide alternate sources. It is uncertain what would constitute ‘adequate’ treatment for this vulnerable group. Just 38% (100/264) of households reported having any form of treatment (either point of use or point of entry device, including chlorination, filtration and UV) on their water supply (Table 1). Furthermore, we did not find any statistically significant association between risk of IID and presence of water treatment (Table 4). One possible explanation is that treatment devices are not maintained or serviced sufficiently by the householder. Further research is required in order to identify a suitable treatment device which can be well maintained by householders.

In conclusion we have shown that IID risk in children under ten years old and drinking from private supplies with evidence of fecal pollution is a serious concern. Indeed the risk of illness is similar to that reported in children from many developing country settings. It is important that households reliant on PWS where children under ten years live or visit are identified and investigated for susceptibility to fecal pollution. In such households, the risk needs to be explained and alternate water sources or well maintained and effective water treatment identified and installed.
